# Multimodal imaging in the diagnosis of uterine polypoid adenomyoma: a case report

**DOI:** 10.1093/bjrcr/uaag029

**Published:** 2026-07-16

**Authors:** Alexandra Jade Davis, Erin Gomez

**Affiliations:** The Russell H. Morgan Department of Radiology and Radiological Science, The Johns Hopkins University School of Medicine, Baltimore, MD 21205, United States; The Russell H. Morgan Department of Radiology and Radiological Science, The Johns Hopkins University School of Medicine, Baltimore, MD 21205, United States

**Keywords:** polypoid adenomyoma, uterine mass, adenomyosis, ultrasound, MRI, case report

## Abstract

Uterine polypoid adenomyoma (UPA) is a rare focal form of adenomyosis that can present as an intracavitary lesion and pose a significant diagnostic challenge, often mimicking more common or concerning entities, such as endometrial polyps or uterine adenosarcoma. We describe the case of a 53-year-old woman who presented with profound anemia (hemoglobin 3.9 g/dL) secondary to acute on chronic heavy vaginal bleeding. Multimodal imaging, including ultrasound and MRI, prompted the initial diagnosis of UPA, later supported by surgical pathology. Ultrasound demonstrated diffusely heterogeneous myometrial echotexture and multifocal “venetian blind” artifact consistent with adenomyosis. MRI revealed an ovoid lesion within the endometrial cavity containing numerous T2 hyperintense cystic foci and imaging characteristics similar to the junctional zone, consistent with a submucosal UPA. Management included blood transfusions, hormone therapy, and ultimately hysterectomy. This case serves to underscore the importance of a combined approach, including both multimodal imaging and histopathology, in guiding the accurate diagnosis and optimal management of UPA. It is also intended to increase awareness of this rare lesion and encourage its consideration in the differential diagnosis of intracavitary uterine masses, as it may have implications for treatment planning.

## Introduction

Uterine polypoid adenomyoma (UPA) is a rare benign uterine lesion that presents as a heterogeneous polypoid mass composed of endometrial glands and stromal tissue with smooth muscle.^1^ It represents a localized form of adenomyosis, also known as an adenomyomatous polyp, and often projects into the endometrial cavity. The clinical presentation is nonspecific, with abnormal uterine bleeding being the most common symptom.^1–3^ Uterine polypoid adenomyoma can be difficult to distinguish from other uterine masses, including endometrial polyps, submucosal leiomyomas, atypical polypoid adenomyomas, and adenosarcoma.^4^ While definitive diagnosis requires histopathological analysis, early diagnosis through accurate identification of UPA on imaging modalities including ultrasound and MRI is valuable for guiding appropriate counseling and management.

## Case presentation

A 53-year-old woman (G5P2032) presented to the emergency department with 5 days of heavy vaginal bleeding (using approximately 10 pads per day), shortness of breath, fatigue, and weakness. She had no abdominal pain or fever and denied recent sexual activity. Past medical history was significant for longstanding menorrhagia, increased over the last 3 years, as well as hypertension and resolved Hepatitis C and HPV infection. Gynecologic history included a benign endometrial biopsy and normal Pap smear with negative HPV in 2014, and remote dilation and curettage.

On arrival, she was alert and oriented, afebrile, with a heart rate of 65-107 bpm, blood pressure of 96-113/51 mmHg, and an oxygen saturation of 96%-100% on room air. Laboratory evaluation was significant for a hemoglobin of 3.9 g/dL (normal 12-16 g/dL) confirmed with recheck, white blood cell count of 27.1 × 10^9^/L (normal 5-10 × 10^9^/L), platelets 567 × 10^9^/L (normal 150-400 × 10^9^/L), and a negative β-hCG. Speculum examination revealed active vaginal bleeding with passage of clots.

Transabdominal and transvaginal ultrasound demonstrated an enlarged uterus with thickened, heterogeneous myometrium consistent with adenomyosis (Figure 1). No fibroids were visualized. Prior outside imaging from 2012 and 2008 had also indicated adenomyosis. MRI of the pelvis without contrast demonstrated a significantly enlarged uterus with a diffusely thickened junctional zone containing scattered masslike foci. An ovoid mass measuring 6.2 × 2.2 × 4.0 cm was visualized in the inferior aspect of the endometrial cavity with imaging features similar to those seen in the junctional zone, including numerous T2 hyperintense cystic foci, suggestive of UPA (Figure 2).

**Figure 1:**
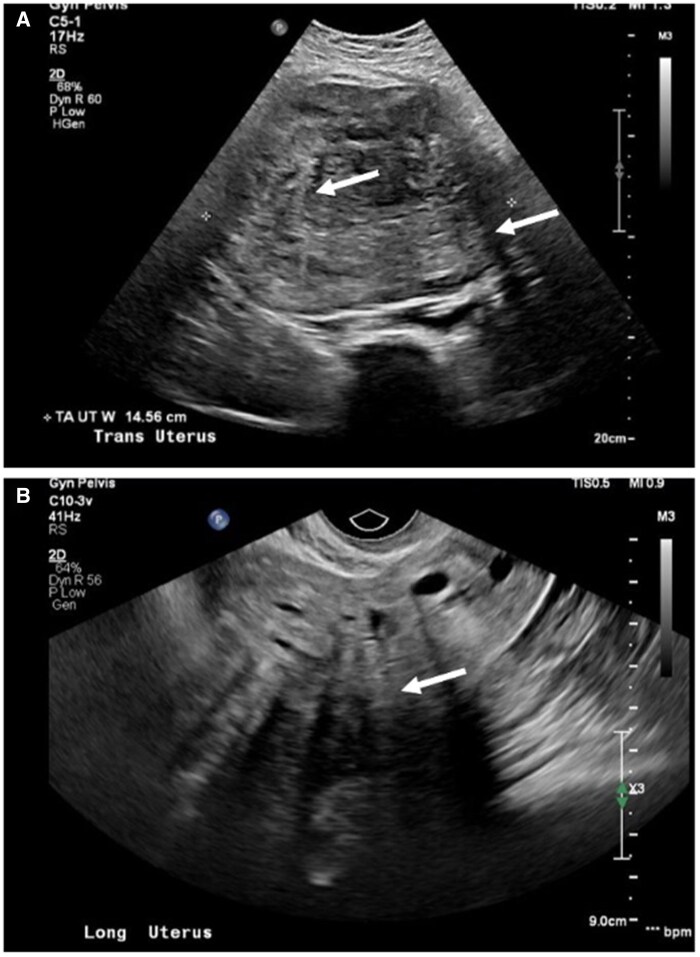
Ultrasound findings. Grayscale transabdominal (A) and transvaginal (B) ultrasound images of the pelvis demonstrating a markedly enlarged uterus and thickened myometrium with diffusely heterogeneous myometrial echotexture and multifocal “venetian blind” artifact (arrows) consistent with adenomyosis.

**Figure 2:**
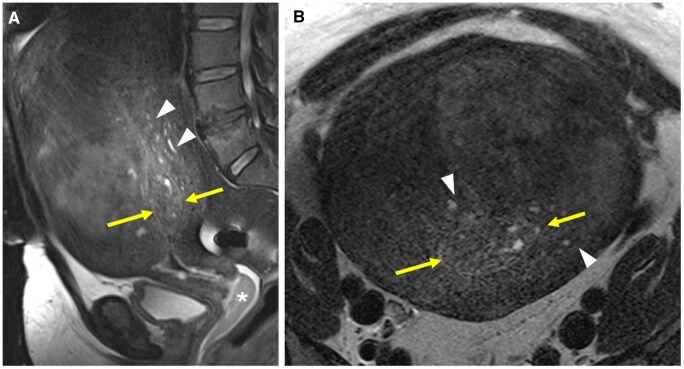
MRI findings. Sagittal (A) and axial (B) T2-weighted images of the pelvis demonstrating marked enlargement of the uterus with heterogeneous myometrial echotexture. The junctional zone is ill-defined and thickened and contains numerous T2 hyperintense cystic foci (arrowheads). An ovoid lesion (arrows) measuring 6.2 × 2.2 × 4.0 cm with similar imaging features to the junctional zone is present and extends into the endometrial cavity at the level of the lower uterine segment. Blood products are noted in the vagina (asterisk).

The patient was admitted to the ICU for profound symptomatic anemia, receiving 6 units of packed red blood cells and intravenous iron, improving her hemoglobin to 8.6 g/dL. Vaginal bleeding was significantly reduced following initiation of progestin therapy (medroxyprogesterone 20 mg 3 times daily). Endometrial biopsy and Pap smear were performed; both showed benign results. The patient was discharged on day 2 of her hospitalization in stable condition.

She subsequently elected to undergo total hysterectomy for definitive treatment. Surgical pathology identified a focus with multiple areas of adenomyosis, with a submucosal and possibly polypoid configuration, as well as leiomyomas showing degenerative changes. Further assessment was limited by specimen morcellation and resulting loss of tissue architecture, preventing accurate determination of the precise locations of the masses.

## Discussion

Adenomyosis is a common benign gynecologic condition characterized by the presence of endometrial glands and stroma within the myometrium, which cause inflammation and can result in a wide spectrum of symptoms, most frequently menorrhagia, dysmenorrhea, and chronic pelvic pain.^4^ Risk factors for adenomyosis include increased exposure to estrogen, eg, due to high parity, as well as endometrial damage, such as prior uterine surgery.^5^ While adenomyosis typically manifests as a diffuse process, it can also present more focally as a discrete mass known as an adenomyoma. When pedunculated and/or intracavitary in location, this is referred to as a UPA. These lesions most commonly arise in the uterine corpus, followed by the fundus and lower uterine segment, with rarer occurrences in the cervix.^1–3^

Transvaginal ultrasound is an excellent initial imaging modality for differentiating endometrial masses. While sonographic features do overlap with other uterine polypoid lesions, findings most suggestive of UPA include heterogeneous echogenicity, the presence of multiple cysts, hemorrhagic foci, posterior shadowing, and coexistence of adenomyosis.^6^ While cystic spaces are the characteristic appearance, UPAs can be solid, cystic, or mixed lesions. The heterogeneous myometrial tissue may create a distinctive posterior shadowing pattern referred to as a “venetian blind” appearance, characterized by linear hypoechoic striations.^7^

MRI is a valuable complementary imaging modality, offering higher sensitivity than ultrasound for identifying focal lesions such as UPAs in the background of adenomyosis, and for differentiating them from leiomyomas.^8^ MRI features characteristic of UPA include isointensity with the myometrium, multiple T2 hyperintense foci (corresponding to ectopic endometrial glands), lack of diffusion restriction, and postcontrast enhancement similar to or less than that of the myometrium.^4^^,^^8^ Uterine polypoid adenomyoma also demonstrates a low choline peak on MR spectroscopy, a finding consistent with its benign character.^9^

Uterine polypoid adenomyoma may enter the differential diagnosis when evaluating intracavitary uterine lesions, alongside entities such as endometrial polyps, submucosal leiomyomas, and adenosarcoma. While histopathological evaluation is necessary for definitive diagnosis, multimodal imaging can offer meaningful insight into differentiating these entities (Table 1). Endometrial polyps are often distinguishable sonographically, as they contain cystic areas only rarely, and their solid components are hyperechoic to the myometrium, as opposed to isoechoic in UPA.^4^^,^^6^ Endometrial polyps also demonstrate characteristic “stalk-like” vascularity on ultrasound. MRI is key for accurately identifying adenosarcoma, which has high signal intensity on diffusion-weighted imaging with low ADC, unlike UPA, which does not restrict diffusion, as well as a high choline peak on MR spectroscopy, suggestive of its malignant nature.^9^ Submucosal leiomyomas, potentially the closest mimic to UPAs, also have several distinguishing features on imaging, including that they are hypoechoic to myometrium, better marginated from myometrium, more commonly calcified, and have only rare cystic changes associated with degeneration.^4^^,^^6^^,^^7^ On MRI, leiomyomas are classically diffusely T2 hypointense, although hyaline degeneration may result in increased T2 signal internally.

**Table 1:** Characteristic imaging features.

Characteristic features		Uterine polypoid adenomyoma	Endometrial polyp	Submucosal leiomyoma	Uterine adenosarcoma
US	Echogenicity relative to myometrium	Heterogeneous	Hyperechoic	Hypoechoic	Heterogeneous
	Solid/cystic	Multiple cysts	Solid, only rarely cystic	Solid, rare degenerative cystic changes	Multiple cysts
	Hemorrhagic foci	Present	Absent	Absent	Present
	Margination	Poorly marginated from myometrium	Limited to endometrium	Well marginated from myometrium	May be limited to endometrium or invading myometrium
	Other	Background adenomyosis	“Stalk-like” vascularity	Calcification	Necrotic foci
MRI	T1 signal intensity relative to myometrium	Isointense	Isointense to hypointense	Isointense	Isointense
	T2 signal intensity	Heterogeneous with hyperintense foci	Hyperintense centrally and hypointense peripherally	Diffusely hypointense (degenerative changes show increased signal internally)	Heterogeneous with hyperintense foci
	DWI	Low	Low	Low	High
	ADC	High	High	High	Low
	Choline peak	Low	Low	Low	High

The most relevant distinguishing features on ultrasound and MRI are given for multiple intracavitary uterine lesions.

The clinical presentation can be similar across these lesions, often involving abnormal uterine bleeding or bulk-related symptoms, but management and prognosis can differ significantly. Treatment for UPA is typically hysteroscopic polypectomy, especially when fertility preservation is desired, though hysterectomy is also a definitive option.^1^^,^^5^ Preoperative diagnosis of UPA is highly relevant for perioperative planning and counseling. For one, UPA has a higher rate of recurrence following conservative surgery than other entities such as endometrial polyps.^5^ UPAs may also present an additional surgical challenge, as the border between lesion and myometrium is less well-defined than in other masses such as leiomyomas, and the presence of cystic lesions makes manipulation more difficult, leading to more complicated dissection and excision.^10^

Diagnostic uncertainty is well recognized in UPA. Although the Morphological Uterus Sonographic Assessment (MUSA) group has established standardized consensus criteria for adenomyosis and adenomyomas on ultrasound, there is currently no equivalent, widely adopted classification system for MRI.^4^ Distinguishing between intracavitary uterine lesions based solely on radiological findings remains difficult, and many entities require further histopathological evaluation. While histopathology remains the definitive diagnostic gold standard, studies have found that in 21%-35% of UPA cases sent for consultation, referring pathologists expressed concern for adenosarcoma.^1^^,^^2^ Additional diagnostic difficulty arises in that multiple lesions may also coexist. For example, concomitant leiomyomas are noted in 30%-47% of patients with UPA.^2^^,^^3^ Given this overlap and the clinical significance of accurate lesion identification, diagnosis is achieved most effectively by correlation of clinical, histopathological, and imaging findings.

## Conclusion

Uterine polypoid adenomyoma is a rare but important inclusion in the differential diagnosis of intracavitary uterine masses, especially when imaging reveals a pedunculated lesion with internal cystic or hemorrhagic components in the context of adenomyosis. Gaps still exist in the establishment of standardized imaging-based diagnostic criteria for UPA, contributing to its ongoing diagnostic challenge. Accurate diagnosis is enhanced with the integrated use of imaging modalities, including ultrasound and MRI, with both clinical and histopathological data, supporting timely and appropriate management.

## Learning points

- Uterine polypoid adenomyoma (UPA) should be considered in the differential diagnosis of intracavitary uterine masses, particularly when a polypoid lesion with cystic or hemorrhagic components is identified in the setting of adenomyosis.
- Multimodal imaging with ultrasound and MRI can improve diagnostic confidence by identifying characteristic features that help distinguish UPA from other intracavitary uterine lesions.
- Preoperative diagnosis of UPA is important for appropriate counseling and surgical planning, given its association with higher recurrence rates and more challenging surgical excision.
